# A New Method for Abrin Detection Based on the Interaction between Target Molecules and Fluorescently Labeled Aptamers on Magnetic Microspheres

**DOI:** 10.3390/ma15196977

**Published:** 2022-10-08

**Authors:** Zhiwei Liu, Zhaoyang Tong, Yuting Wu, Bing Liu, Shasha Feng, Xihui Mu, Jiang Wang, Bin Du, Jianjie Xu, Shuai Liu

**Affiliations:** State Key Laboratory of NBC Protection for Civilian, Beijing 102205, China

**Keywords:** abrin, aptamer, QSAR model, magnetic microspheres, fluorescence quenching, detection

## Abstract

A quantitative structure–activity relationship (QSAR) model for the structure and affinity of abrin aptamers was established. A higher affinity abrin aptamer based on the established QSAR model was screened by site-directed mutagenesis. The fluorescence quenching effect between magnetic microspheres and fluorescent molecules was studied for the first time. A new method for abrin detection based on the interaction between target molecules and fluorescently labeled aptamers on magnetic microspheres was developed, with the detection limit of 5 ng mL^−1^. This method can overcome the influence of complex environmental interferents in abrin detection and can meet the analysis requirements for simulated samples such as water, soil, and food.

## 1. Introduction

Abrin is a type II ribosome-inactivating protein present in seeds from the plant *Abrus precatorius*, which is highly toxic to eukaryotic cells [1,2]. Abrin is similar to ricin in its sequence and structure level, but its toxicity is stronger than ricin. Because of its wide distribution and high lethality, abrin has become a potential ideal terrorism biological warfare agent [3,4]. Therefore, research focused on the rapid detection and specific recognition of abrin should be paid more attention. Currently, the detection of abrin is mainly based on the immunoassay method [5,6,7,8,9,10], which can realize trace detection. However, it is relatively difficult to generate and prepare the antibodies, especially for virulent toxins. Therefore, it is of great significance to find a ligand molecule that has a specific reaction to abrin and is relatively easy to prepare.

Aptamers are single-stranded DNA/RNA that can form specific tertiary structures and bind to target molecules in a manner similar to antibodies. Based on the technology of the systematic evolution of ligands by exponential enrichment (SELEX), aptamers with a high affinity to target molecules can be screened from random single strand nucleic acid sequence libraries. Aptamers have the advantages of a high affinity and specificity, a wide range of target molecules, and easy preparation. They have been widely used in the detection of small molecules [11,12,13,14], proteins [15,16,17], viruses [18,19,20,21], cells [22,23,24,25], and bacteria [26,27,28,29]. There are few reports on the use of aptamers to detect abrin [2,30]. Although the specific detection of abrin at the nM level has been achieved, there is still room for improvement. In previous reports, abrin detection requires special optical reagents capable of inserting double stranded DNA and strong oxidizing reagents (such as hydrogen peroxide). Therefore, it is significant to establish a milder detection method.

Magnetic microspheres play an important role in the enrichment and separation of biomolecules and are widely used as the carrier of capture probes in biomolecular detection [31,32]. When we analyzed the common factors affecting fluorescence in biological detection, we found that the presence of magnetic microspheres in solution would greatly reduce the fluorescence signal of fluorescent substances. We speculate that magnetic microspheres can be used as fluorescence quenching materials to detect target molecules, which has not been reported yet.

In our laboratory, fifteen aptamers with different sequences and affinities for abrin were screened by SELEX. In this study, the four nucleotides structure was used to characterize the abrin aptamer structure according to the arrangement of nucleotides in the aptamers. A quantitative structure–activity relationship (QSAR) model was established between the structure and affinity of the abrin aptamers. A higher affinity aptamer based on the established QSAR model was screened by site-directed mutagenesis. Then the fluorescence quenching effect between magnetic microspheres and fluorescent molecules was studied for the first time. Finally, a new method for abrin detection based on the interaction between target molecules and fluorescently labeled aptamers on magnetic microspheres was developed.

## 2. Materials and Methods

### 2.1. Reagents and Apparatus

Fifteen aptamers with different affinities for abrin are shown in Table S1. Biotinylated single-stranded DNA (ssDNA) (5′-Bio-ACTGACTGCTGAACA-3′), Alexa405/ROX-labeled ssDNA (3′-TGACTGACGACTTGT-Alexa405/ROX-5′), a biotinylated abrin aptamer (5′-Bio-aptamer-3′), and a biotin and ROX labeled abrin aptamer (5′-Bio-aptamer-ROX-3′) were synthesized by Beijing Xing Fangyuan Biotechnology Co., Ltd. (Beijing, China). Abrin and ricin were purchased from Beijing Hapten and the Protein Biomedical Institute (Beijing, China). Streptavidin-coated magnetic microspheres (Dynabeads^TM^ M-280 Streptavidin) were purchased from Invitrogen, Life Technologies Corp. (Carlsbad, CA, USA). Bovine serum albumin (BSA) was purchased from Solarbio Life Sciences (Beijing, China).

The affinity and specificity were determined by an Octet K2 molecular interaction analysis system (Sartorius AG, Göttingen, Germany). The affinity of the aptamers was characterized by an equilibrium dissociation constant (K_D_). The Bio-Layer Interferometry (BLI) sensor used to determine the K_D_ of the aptamer was purchased from Sartorius AG (Göttingen, Germany): High Precision Streptavidin sensor (sensor type: SAX2.0). The fluorescence was determined by a multifunctional enzyme-labeled instrument (TECAN spark, Männedorf, Switzerland). The magnetic separation was performed by magnetic frames (Promega Company, Madison, WI, USA). Stepwise regression was realized by IBM SPSS Statistics 19 (IBM, Armonk, NY, USA).

### 2.2. QSAR Model of Abrin Aptamers and Virtual Screening of Abrin Aptamer

Referring to our previous report [33], the structure of four nucleotides is represented by three principal components (Table S2), which are obtained by a principal component analysis of the two-dimensional property parameters of the four nucleotides. The four nucleotides structure was used to characterize the abrin aptamer structure according to the arrangement of nucleotides in the aptamers. Based on the stepwise regression method, the structural characterization variables (independent variables) that have a significant impact on abrin aptamer affinity (dependent variables) were screened, and the regression model between the structure and affinity of abrin aptamers was established. Based on the QSAR model of abrin aptamers, stepwise site-directed mutagenesis for abrin-2 (it had the highest affinity in the fifteen abrin aptamers) was performed to improve the affinity. The smaller the K_D_ value, the greater the affinity. The specific steps of K_D_ determination are shown in the Supplementary Materials.

### 2.3. Study on the Fluorescence Quenching Effect between Magnetic Microspheres and Fluorescent Molecules

The fluorescence quenching effect between the streptavidin-coated magnetic microspheres and fluorescent molecules (Alexa405 and ROX) was studied. Streptavidin-coated magnetic microspheres (10 mg mL^−1^) were washed with PBST-B prepared in our lab (0.01 M PBS, containing 0.02% Tween-20 and 0.5% BSA, pH 7.4) and then were added 1 μM of biotinylated ssDNA (15 nucleotides, the theoretical length is about 5.3 nm). The reaction was rotated at room temperature for 1 h. After magnetic separation, 1 uM of fluorescently labeled complementary ssDNA (15 nucleotides) was added and combined to the streptavidin-coated magnetic microspheres by a DNA base complementary matching principle. Then, the fluorescence of fluorescent molecules bound to the streptavidin-coated magnetic microspheres at a distance of about 5.3 nm was measured. The fluorescent molecule which had a good fluorescence quenching effect with the streptavidin-coated magnetic microspheres was selected to label the abrin aptamer. The affinity and specificity of the fluorescently labeled abrin aptamer was determined. The specific steps of determination are shown in the Supplementary Materials.

### 2.4. Detection of Abrin Based on the Interaction between Target Molecules and Fluorescently Labeled Aptamers on Magnetic Microspheres

Streptavidin-coated magnetic microspheres (10 mg mL^−1^) were washed with PBST-B and then added to 5 nM, 50 nM, 250 nM, 500 nM, 1 μM, 2 μM, and 8 μM of biotin and fluorescently labeled abrin aptamer, respectively. Biotin and fluorescently labeled abrin aptamer were attached to the streptavidin-coated magnetic microspheres through biotin avidin interaction. The reaction was rotated at room temperature for 1 h. After magnetic separation, the fluorescence of the magnetic microspheres bound with the fluorescently labeled aptamer was measured and the optimal immobilized concentration of the biotin and fluorescently labeled abrin aptamer was determined according to the measured fluorescence signals.

Different concentrations of abrin reacted with the fluorescently labeled aptamer bound on the magnetic microspheres for 1 h. After magnetic separation, the fluorescence of the fluorescently labeled aptamer on the magnetic microspheres was determined after magnetic separation. To evaluate the specificity of the method, possible interfering substances such as ricin and BSA were also detected. To investigate whether the assay could overcome the effect of complex environmental interference on abrin detection and could be used for practical assays, samples of river water, fertilized soil, and butter biscuit containing 20 ng mL^−1^ of abrin were prepared. The preparation process of the simulated samples is referred to in our previous report [34]. These samples were assayed using the same method, and the recoveries, relative standard deviations (RSDs), and other necessary indicators were calculated.

## 3. Results

**Figure 1:**
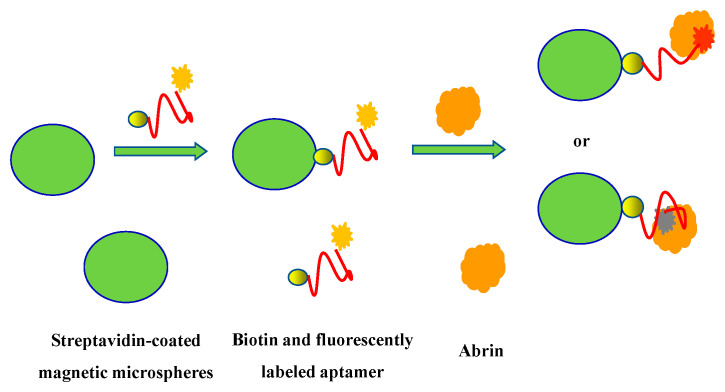
The principle of abrin detection based on the interaction between target molecules and fluorescently labeled aptamers on magnetic microspheres.

The principle of abrin detection is shown in Figure 1. According to Förster’s theory, the efficiency of the fluorescence resonance energy transfer is relevant to the lifetime of the donor in the absence of the acceptor and the sixth power of the distance between the donor and the acceptor [35]. Therefore, the change in the donor–acceptor distance will significantly affect the fluorescence intensity. The combination of abrin and the fluorescently labeled aptamer on the magnetic microspheres leads to the conformation change in the abrin aptamer, the distance change between fluorescent molecules and magnetic microspheres, and finally the fluorescence change in the fluorescent molecules.

### 3.1. QSAR Model of Abrin Aptamers and Virtual Screening of Abrin Aptamer

The stepwise regression results are shown in Tables S3–S6. Six variables that had a significant impact on abrin aptamer affinity were screened. The six variable multiple linear regression equation is as follows:

y = 0.078 + 0.093 x_31_ − 0.091 x_27_ − 0.086 x_76_ + 0.062 x_58_ + 0.069 x_6_ − 0.027 x_54_ (1)

Based on the six variable multiple linear regression equation, site-directed mutagenesis for the abrin aptamer was performed. Then, three new abrin aptamers were designed (Table 1), named abrin-M1, abrin-M2, and abrin-M3, respectively. The secondary structure of each aptamer predicted by the software RNA structure is shown in Figure S1.

**Figure 2:**
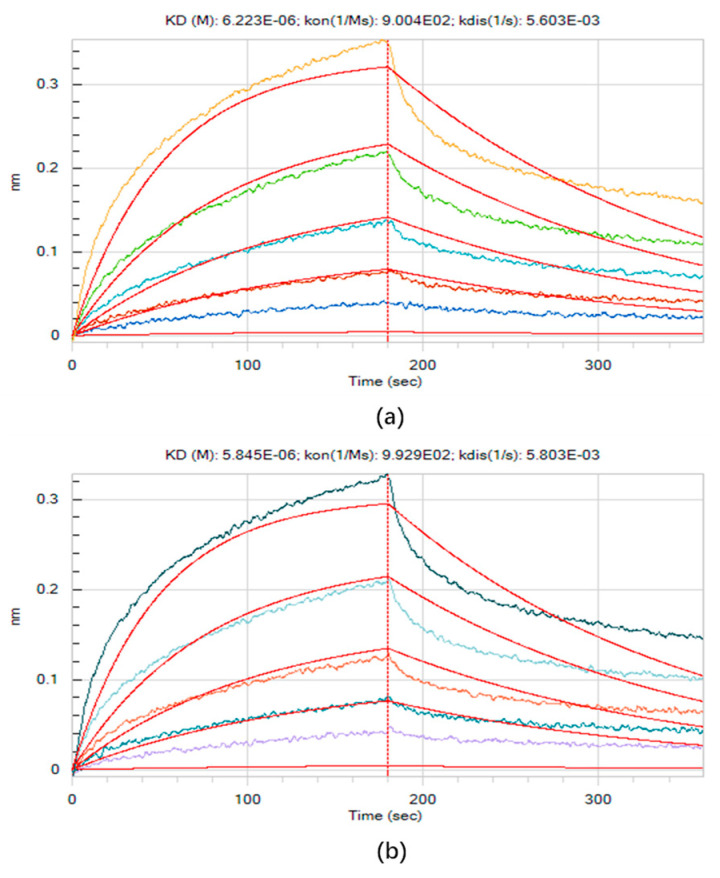
(**a**) K_D_ determination results of abrin-2 with abrin; (**b**) K_D_ determination results of abrin-M1 with abrin.

The affinity of the virtual screened aptamers was characterized by K_D_. The preliminary measurement results are shown in Figure S2. The K_D_ of the biotinylated abrin-2, abrin-M1, abrin-M2, and abrin-M3 combined with abrin was 1.818 × 10^−6^, 1.758 × 10^−6^, 1.971 × 10^−6^, and 2.064 × 10^−6^, respectively. The aptamer with the highest affinity was abrin-M1. The accurate measurement results are shown in Figure 2 (The response signal increased with the increase of abrin concentration). The K_D_ of the biotinylated abrin-2 and abrim-M1 combined with abrin was 6.223 × 10^−6^ (Figure 2a) and 5.845 × 10^−6^ (Figure 2b), respectively. The affinity of abrin-M1 was higher than abrin-2.

### 3.2. Study on the Fluorescence Quenching Effect between Magnetic Microspheres and Fluorescent Molecules

**Figure 3:**
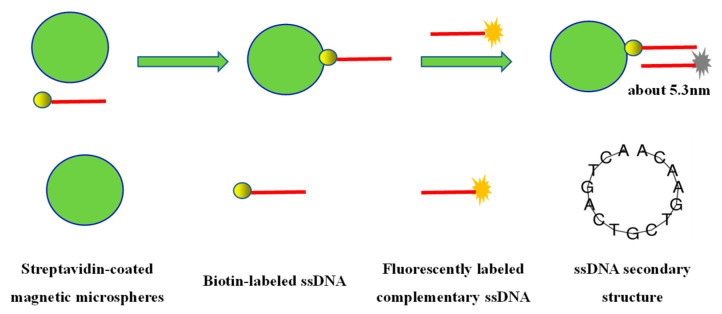
The research idea of fluorescence quenching effect between magnetic microspheres and fluorescent molecules.

Generally speaking, when the distance between the donor and the acceptor is within 10 nm, a strong fluorescence resonance energy transfer will occur. The research idea of the fluorescence quenching effect between magnetic microspheres and fluorescent molecules is shown in Figure 3. Two completely complementary ssDNA with a length of 15 nucleotides (theoretical length is about 5.3 nm) were designed. One ssDNA was labeled with biotin and the other ssDNA was labeled with fluorescent molecules (Alexa405 or ROX). Biotin labeled ssDNA binds to the streptavidin-coated magnetic microspheres through a biotin–avidin interaction. Then, the complementary ssDNA labeled with fluorescent molecules is combined with the streptavidin-coated magnetic microspheres through base complementation. The fluorescence quenching effect of the streptavidin-coated magnetic microspheres and fluorescent molecules at about 5.3 nm was studied.

**Figure 4:**
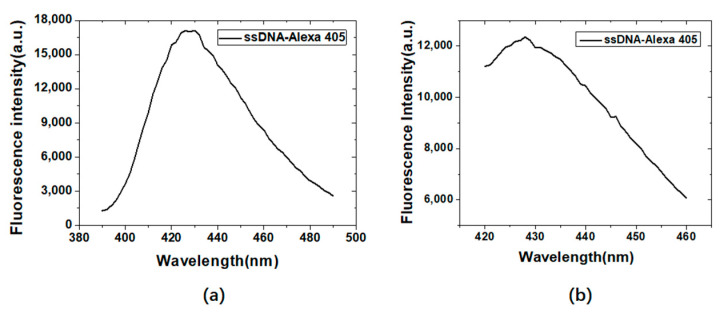
Fluorescence emission scanning results of Alexa405 labeled ssDNA at excitation wave-lengths of 300 nm (**a**) and 370 nm (**b**).

Figure 4a,b are the fluorescence emission scanning results of Alexa405 labeled ssDNA at excitation wavelengths of 300 nm and 370 nm, respectively.

**Figure 5:**
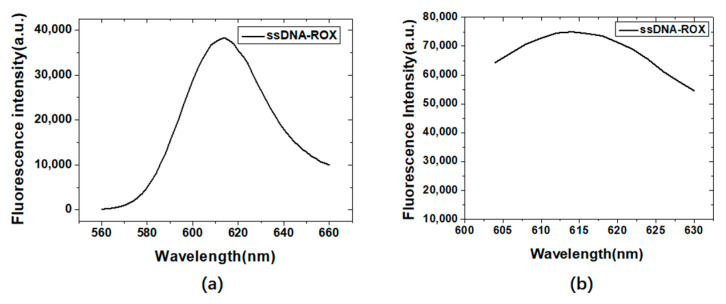
Fluorescence emission scanning results of ROX labeled ssDNA at excitation wavelengths of 500 nm (**a**) and 550 nm (**b**).

Figure 5a,b are the fluorescence emission scanning results of ROX labeled ssDNA at excitation wavelengths of 500 nm and 550 nm, respectively. The maximum fluorescence emission wavelengths of the Alexa405 labeled ssDNA and the ROX labeled ssDNA were 428 nm and 614 nm, respectively, which did not depend on the excitation wavelength. The larger the excitation wavelength, the stronger the fluorescence intensity. Therefore, in subsequent studies, the excitation wavelengths of Alexa405 and ROX were determined to be 370 nm and 550 nm, respectively.

**Figure 6:**
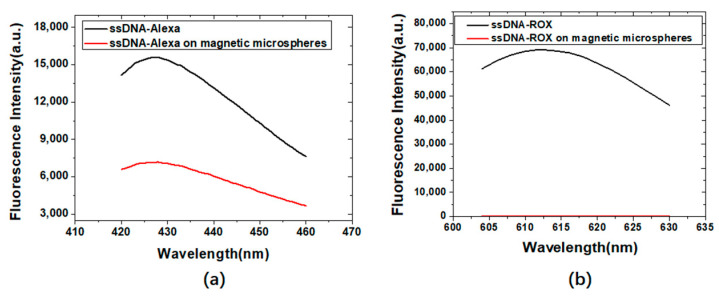
Fluorescence measurement results of Alexa405 (**a**) or ROX (**b**) labeled ssDNA before and after binding to streptavidin-coated magnetic microspheres.

The fluorescence measurement results of Alexa405 (a) or ROX (b) labeled ssDNA before and after binding to streptavidin-coated magnetic microspheres are shown in Figure 6. Streptavidin-coated magnetic microspheres had a good fluorescence quenching effect on ROX. ROX was selected to label the abrin aptamer.

**Figure 7:**
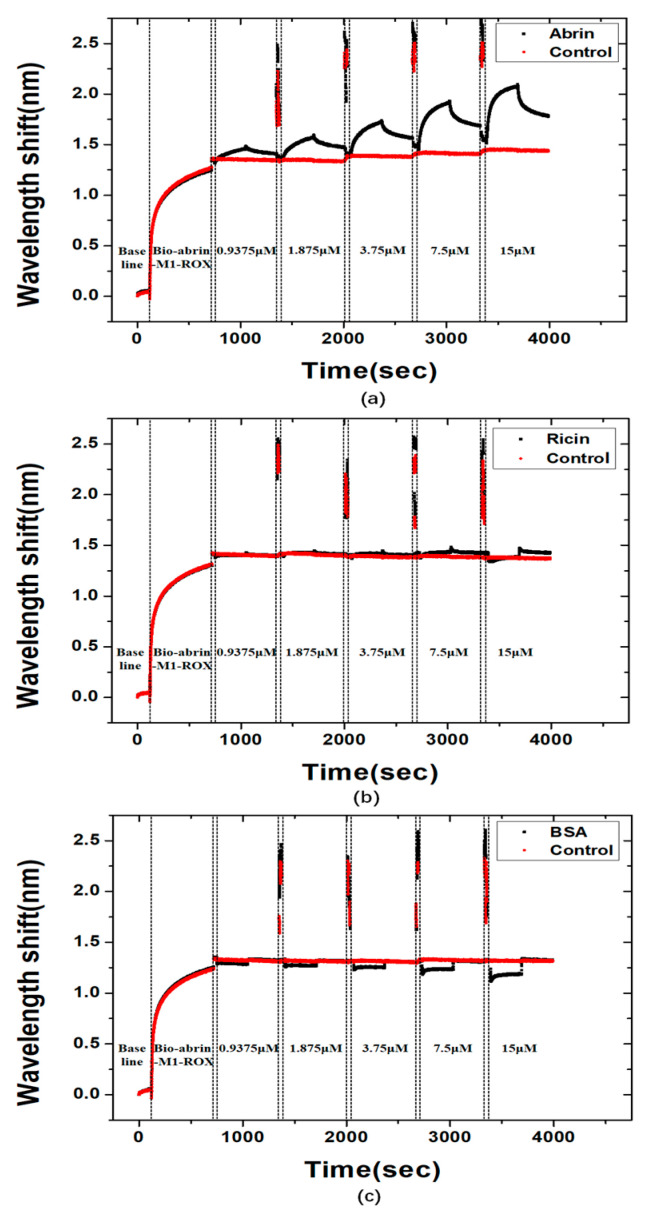
Association and dissociation curves of the ROX labeled abrin-M1 with different concentrations of abrin (**a**), ricin (**b**), and BSA (**c**).

The association and dissociation curves of the ROX labeled abrin-M1 with different concentrations of abrin (Figure 7a), ricin (Figure 7b), and BSA (Figure 7c) are shown in Figure 7. The results showed that the ROX labeled abrin-M1 had a good specificity to abrin, which showed little associations to ricin (ricin is similar to abrin in sequence and structure level) and BSA. The K_D_ of the ROX labeled abrin-M1 combined with abrin was 2.062 × 10^−6^ (Figure S3). The results showed that ROX labeling did not decrease the binding affinity of abrin-M1 to abrin.

### 3.3. Detection of Abrin Based on the Interaction between Target Molecules and Fluorescently Labeled Aptamers on Magnetic Microspheres

**Figure 8:**
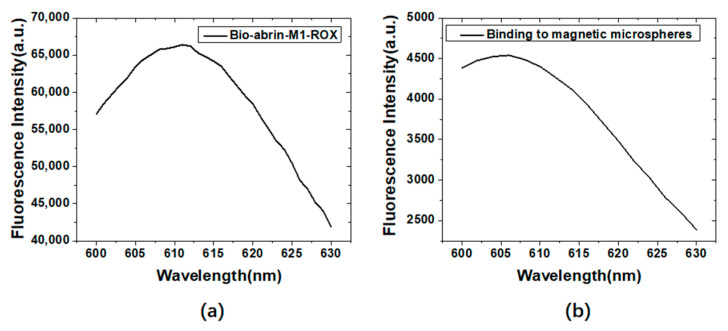
Fluorescence measurement results of ROX labeled abrin-M1 before (**a**) and after (**b**) binding to streptavidin-coated magnetic microspheres.

Figure 8a,b are the fluorescence measurement results of ROX labeled abrin-M1 before (a) and after (b) binding to the streptavidin-coated magnetic microspheres. The maximum fluorescence emission wavelengths were 612 nm and 606 nm, respectively. Compared with ROX labeled ssDNA, the maximum fluorescence emission wavelength blue shifted by 2 nm and 8 nm.

**Figure 9:**
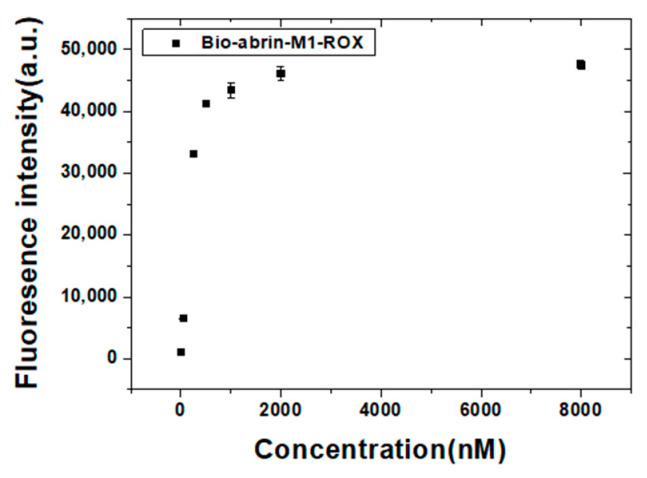
The fluorescence intensity of streptavidin-coated magnetic microspheres combined with different concentrations of biotin and ROX labeled abrin-M1.

Figure 9 shows the fluorescence intensity of the streptavidin-coated magnetic microspheres combined with different concentrations of biotin and ROX labeled abrin-M1. The results showed that the addition of more than 2 μM of biotin and ROX labeled abrin-M1 enhanced the fluorescence intensity a little. After biotin and ROX labeled abrin-M1 were added at more than 500 nM, the increase of the fluorescence intensity was significantly slowed down. Considering the cost and fluorescence intensity, the optimal immobilized concentration of biotin and ROX labeled aptamers was determined to be 500 nM.

**Figure 10:**
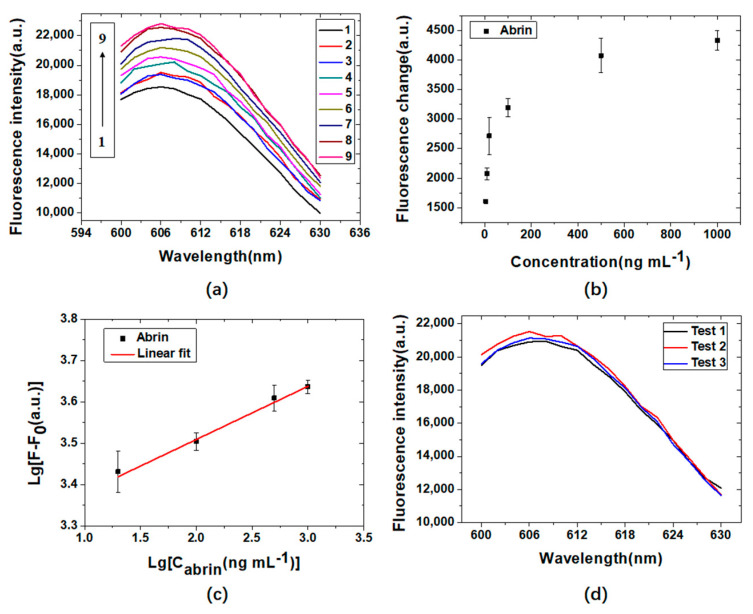
(**a**) Abrin detection results based on the interaction between target molecules and fluorescently labeled aptamers on magnetic microspheres (1–9 are: 0.01 M PBS, 1000 ng mL^−1^ BSA, 100 ng mL^−1^ ricin, 5 ng mL^−1^ abrin, 10 ng mL^−1^ abrin, 20 ng mL^−1^ abrin, 100 ng mL^−1^ abrin, 500 ng mL^−1^ abrin, and 1000 ng mL^−1^ abrin); (**b**) the fluorescence changes induced by the addition of different concentrations of abrin; (**c**) logarithmic linear relationship between the concentrations of abrin and the fluorescence changes; (**d**) the fluorescence curves of three reproducibility tests at a concentration of 20 ng mL^−1^.

The abrin detection results based on the interaction between the target molecules and fluorescently labeled aptamers on magnetic microspheres are shown in Figure 10a. Figure 10b shows the fluorescence changes induced by the addition of different concentrations of abrin. The criterion for determining the detection limit is that the fluorescence of the sample is greater than the negative control (PBS) fluorescence + 3 × standard deviation (SD) [34]. The mean value of the fluorescence measured by the three negative controls was 18,532 (a.u.), and the SD was 208 (a.u.). The detection threshold calculated according to the above criteria was 19,156 (a.u.). Then the detection limit of abrin was determined to be 5 ng mL^−1^. A logarithmic linear relationship between the fluorescence change (F − F_0_) and the concentration of abrin (C_abrin_) in the range of 20 ng mL^−1^ to 1000 ng mL^−1^ was obtained (Figure 10c) with the linear regression equation of Lg (F − F_0_) = 0.126 Lg (C_abrin_) + 3.257 (N = 4, R = 0.994, *p* < 0.005). Figure 10d shows the three test results of 20 ng mL^−1^ of abrin. The fluorescence intensities were 21195 ± 317 (a.u.) (n = 3). Its relative standard deviation was 1.5%, which indicated a good repeatability. The fluorescence changes of ricin (100 ng mL^−1^) and BSA (1000 ng mL^−1^) were much less than that of abrin (100 ng mL^−1^), which indicated a high specificity.

As shown in Table 2, simulated samples which contained abrin in river water, fertilized soil, and butter biscuit were examined, showing a recovery ratio of 84.5~106.5% and a relative standard deviation (RSD) of 5.16~9.90%. This method met the requirements for the analysis of the simulated samples above, showing acceptable recovery rates and reproducibility.

## 4. Discussion

**Figure 11:**
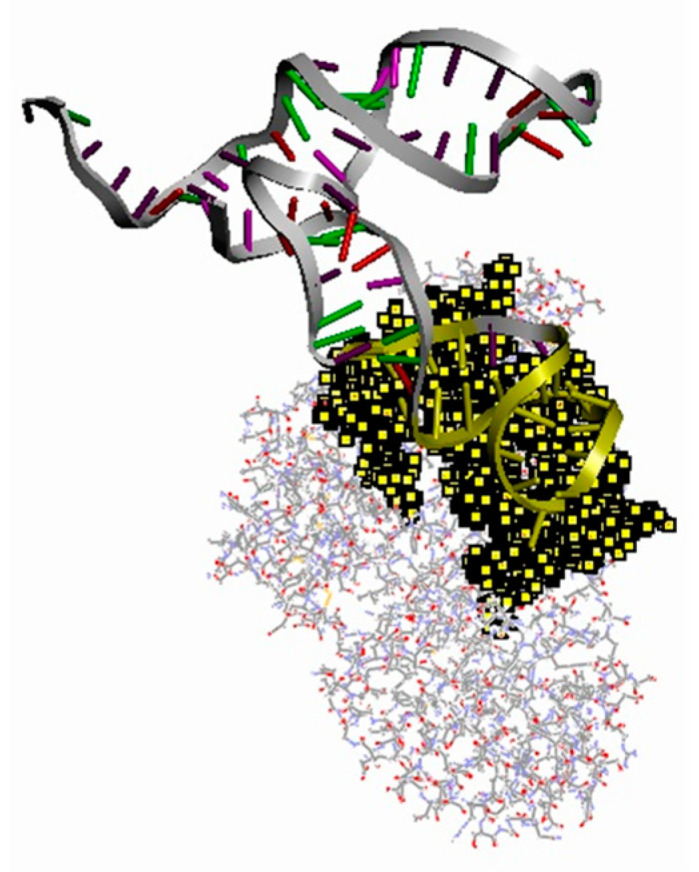
Docking results of abrin-M1 and abrin (the docking interface is shown in yellow).

In order to verify whether the combination of abrin and abrin-M1 could increase the distance between the ROX and magnetic microspheres, the molecular docking calculation of abrin (PDB code: 1ABR) and abrin-M1 was carried out by referring to the reported method [33,36]. The docking results, and the binding interface of abrin-M1 and abrin, are shown in Figure 11. The binding site is shown in Table S7. According to the molecular docking results, the combination of abrin and abrin-M1 will exert a drag force on abrin-M1, causing the 3′-terminal and 5′-terminal of abrin-M1 to move away from each other, thus the distance between the 3′-terminal labeled ROX and the 5′-terminal bound magnetic microspheres is increased. The molecular docking results are in a good agreement with the experimental results.

Studies have shown that when the distance between the donor and the acceptor is between 5 nm and 30 nm, the fluorescence intensity changes significantly with the distance between them [37]. In this study, the number of nucleotides in the aptamer was 78. If all nucleotides of the aptamer are completely perpendicular to the surface of the magnetic microspheres, the distance between the fluorescent molecule labeled at the 3′-terminal of the aptamer and the surface of the magnetic microspheres is about 27 nm~28 nm. However, the aptamer folds to form a tertiary structure. Therefore, the distance between the fluorescent molecules at the 3′-terminal of the aptamer and the surface of the magnetic microspheres must be less than 27 nm~28 nm. After connecting the 3′-terminal fluorescently labeled aptamer to the surface of the magnetic microspheres, strong fluorescence can still be detected, indicating that the distance between the fluorescent molecules at the 3′-terminal of the aptamer and the surface of the magnetic microspheres is greater than 5.3 nm. That is, the distance between the fluorescent molecule at the 3′-terminal of the aptamer and the surface of the magnetic microspheres is 5.3 nm to 27 nm~28 nm. Within this distance range, the fluorescence intensity varies significantly with the distance between the donor and the acceptor. Therefore, after the aptamer binds to the abrin, due to the conformational change of the aptamer, the distance between the fluorescent molecule and the magnetic microspheres changes, which will lead to a significant change in the fluorescence intensity of the fluorescent molecule.

The detection limit (5 ng mL^−1^ ≈ 0.077 nM) of this method is the same order of magnitude as that of the previous aptamer-based methods for abrin detection (their detection limits for abrin were 1 nM and 0.05 nM, respectively) [2,30], but the detection condition is milder and the construction process is simpler.

## 5. Conclusions

In this study, the four nucleotides structure was used to characterize the abrin ap-tamer structure according to the arrangement of nucleotides in the aptamers. A QSAR model for the structure and affinity of the abrin aptamers was established. A higher affinity abrin aptamer based on the established QSAR model was screened by site-directed mutagenesis. The fluorescence quenching effect between magnetic microspheres and fluorescent molecules was studied for the first time. A new method for abrin detection based on the interaction between target molecules and fluorescently labeled aptamers on magnetic microspheres was developed, with the detection limit of 5 ng mL^−1^. This method can overcome the influence of complex environmental interferents in abrin detection and can meet the analysis requirements for simulated samples such as water, soil, and food.

## Figures and Tables

**Table 1:** The sequence of three new aptamers.

Name	Sequence
Abrin-2	5′-TCGCAAGACGGACAGAAGGCTTGTGGTCTTCTTTTGGTG ATGTACTGCCGTTAATGGAGTGTTGGTGGAGCGATTTGT-3′
Abrin-M1	5′-TCGCAAGACGGACAGAAGGCTTGTGGTCCTCTTTTGGTG ATGTACTGCCGTTAATGGAGTGTTGGTGGAGCGATTTGT-3′
Abrin-M2	5′-TCGCAAGACGGACAGAAGGCTTGTGGTCCTCTTTTGGTG ATGTGCTGCCGTTAATGGAGTGTTGGTGGAGCGATTTGT-3′
Abrin-M3	5′-TCGCAAGACGGACAGAAGGCTTGTGGTCCTCTTTTTGTG ATGTGCTGCCGTTAATGGAGTGTTGGTGGAGCGATTTGT-3′

**Table 2:** Determination of the simulated abrin specimens (n = 3).

Sample	Added (ng mL^−1^)	Found (ng mL^−1^)	Recovery Ratio (%)	Relative Standard Deviation (%)
River water	20	21.3 ± 1.1	106.5	5.16
Fertilized soil	20	20.2 ± 2.0	101.0	9.90
Butter biscuit	20	16.9 ± 1.2	84.5	7.10

## Data Availability

Data presented in this study are available on request from the authors.

## Supplementary Materials

The following are available online at https://www.mdpi.com/article/10.3390/ma15196977/s1, Figure S1: Secondary structure of abrin-2 and three new aptamers, Figure S2: K_D_ preliminary determination results of abrin-2 and three new aptamers with abrin, Figure S3: K_D_ determination results of the ROX labeled abrin-M1 with abrin, Table S1: The sequence and affinity of abrin aptamers screened by our laboratory, Table S2: Principal component scores of the four nucleotides, Table S3: Variables Entered/Removed, Table S4: Model Summary, Table S5: ANOVA, Table S6: Coefficients, Table S7: Interaction sites between abrin-M1 and abrin.
