# Supplementary material for: A New Method for Abrin Detection Based on the Interaction between Target Molecules and Fluorescently Labeled Aptamers on Magnetic Microspheres
Source: Materials (Basel). 2022 Oct 8;15(19):6977. doi: 10.3390/ma15196977 (PMC9573059; doi:10.3390/ma15196977)
Supplement: Supplementary file 1 [file materials-15-06977-s001.zip › materials-1925821-supplementary.pdf]

A New Method for Abrin Detection Based on the Interaction between Target Molecules and Fluorescently Labeled Aptamers on Magnetic Microspheres

Zhiwei Liu, Zhaoyang Tong *, Yuting Wu, Bing Liu, Shasha Feng, Xihui Mu, Jiang Wang, Bin Du, Jianjie Xu and Shuai Liu

State Key Laboratory of NBC Protection for Civilian, Beijing 102205, China
* Correspondence: billzytong@126.com

Table S1. The sequence and affinity of abrin aptamers screened by our laboratory.

Name	Sequence	Affinity (characterized by OD_{450}, determined by enzyme-linked immunosorbent assay)
Abrin-1	5′-TCGCAAGACGGACAGAAGGCAGCTTTTTTGTGTTTCT GCTTCAGTCTGCGGGGGGTTGGG GTTGGTGGAGCGATTTGT-3′	1.408
Abrin-2	5′-TCGCAAGACGGACAGAAGGCTTGTGGTCTTCTTTTGG TGATGTACTGCCGTTAATGGAGTGTTGGTGGAGCGATTT GT-3′	1.512
Abrin-3	5′-TCGCAAGACGGACAGAAGCTGATGCTTTGTTTAACTG CTTTATCATTGTACCTAGGGCCG GTTGGTGGAGCGATTTGT-3′	0.666
Abrin-4	5′-TCGCAAGACGGACAGAAGCGGGGTTTTGTATGTTAGT TTATTGTTGGTCATGTTCAGTTGGTTGGTGGAGCGATTTG T-3′	1.288
Abrin-5	5′-TCGCAAGACGGACAGAAGTGGATTACCTTCCTTTGTC TGGTCAGGCCTGTCTCTTTCGTGGTTGGTGGAGCGATTTG T-3′	1.226
Abrin-6	5′-TCGCAAGACGGACAGAAGTGCTGCGGGCCCCTGCTT GGTGTGCAATGCCCTGGGCGTACGGTTGGTGGAGCGATT TGT-3′	1.093
Abrin-7	5′-TCGCAAGACGGACAGAAGGGGTTTTTGTCCAGGTTCA TTATTGTTGTTGAATGTACGATT GTTGGTGGAGCGATTTGT-3′	0.898
Abrin-8	5′-TCGCAAGACGGACAGAAGTGCTGCGGGCCCCTGCTT GGTGTGCAATGCCCTGGGCGTACGGTTGGTGGAGCGATT TGT-3′	0.903

Abrin-9	5′-TCGCAAGACGGACAGAAGGGTGGCTTGTCTTTTTGTG GTTTTTCCGTACCCCCAATCAGT GTTGGTGGAGCGATTTGT-3′	0.730
Abrin-10	5′-TCGCAAGACGGACAGAAGGACGTTTGCGTGGCTTATG CTTTGGCGGGTAATTTGTGGGCG GTTGGTGGAGCGATTTGT-3′	0.700
Abrin-11	5′-TCGCAAGACGGACAGAAGGAAAACAAAAGTTTTATG TGGGTGTGTGCGGTATGGATTTCGGTTGGTGGAGCGATTT GT-3′	0.649
Abrin-12	5′-TCGCAAGACGGACAGAAGCGTCATTCGTGTGTTTTAG TTGTTACTGTAGGCGGATCGGGGGTTGGTGGAGCGATTT GT-3′	0.583
Abrin-13	5′-TCGCAAGACGGACAGAAGCTGTGATCTGTTGTGTTATT TGGGGCCTTCTTTTGCCTATCCGTTGGTGGAGCGATTTGT-3′	0.926
Abrin-14	5′-TCGCAAGACGGACAGAAGTACGTAGGTTAGTTATGTG TTATGATCCTGTTTTGTCCAGGCGTTGGTGGAGCGATTTG T-3′	1.021
Abrin-15	5′-TCGCAAGACGGACAGAAGCATGTTCTACGTGAATACT GGATCAGCTTTCTCTGTCACGCGGTTGGTGGAGCGATTT GT-3′	0.661
BSA	/	0.112

A single-stranded DNA (ssDNA) random library was constructed for abrin aptamers screening by in vitro synthesis. The sequence of the random library is 5′-TCGCAAGACGGACAGAAG -(N)$_{42}$-GTTGGTGGAGCGATTTGT-3′ (N is a random nucleotide which may be A, C, G, and T). The sequences of the PCR-amplified primers are: the upstream primer p1 is 5′-TCGCAAGACG GACAGAAG-3′, the downstream primer p2 is 5′-ACAAATCGCTCCACCAAC-3′. In the process of screening aptamers of abrin by SELEX, abrin aptamers were separated by magnetic beads in our laboratory.

Table S2. Principal component scores of the four nucleotides.

	**A**	**C**	**G**	**T**
C_1	0.287889871	-1.058501257	1.251265726	-0.480654338
C_2	1.445345997	-0.103555766	-0.675927084	-0.665863148
C_3	0.2794537	-1.057757671	-0.476924168	1.255228137

Specific steps of K_D determination of the virtual screened aptamers: biotinylated aptamers were immobilized on the High Precision Streptavidin sensors for 10 min. The sensor modified with abrin aptamer (the virtual screened aptamers or abrin-2) was moved to the sample wells and reacted with 15 μM abrin. The negative control was PBST (0.01 M PBS, containing 0.02% Tween-20, pH 7.4). Step time of the association and dissociation was 3 min, respectively. The affinity was preliminary determined. Then the sensor modified with abrin aptamer (the virtual screened aptamers with higher affinity than abrin-2 or abrin-2) was moved to the sample wells and reacted with 0.9375 μM, 1.875 μM, 3.75 μM, 7.5 μM, and 15 μM abrin respectively (or PBST as the negative control in reference wells). PBST-NaCl (0.01 M PBS, containing 0.02% Tween-20 and 1.5M NaCl, pH 7.4) was used to regenerate the sensors before the next combination of abrin. Step time of the association and dissociation was 3 min, respectively. The affinity was accurate determined.

Specific steps of affinity and specificity determination of the fluorescently labeled abrin aptamer: biotin and fluorescently labeled abrin aptamers were immobilized on the High Precision Streptavidin sensors for 10 min. The sensor modified with abrin aptamer was moved to the sample wells and reacted with 0.9375 μM, 1.875 μM, 3.75 μM, 7.5 μM, and 15 μM abrin respectively (or PBST as the negative control in reference wells). PBST-NaCl was used to regenerate the sensors before the next combination of abrin. Step time of the association and dissociation was 5 min, respectively. Ricin and BSA were used as interfering substances to determine the specificity of the fluorescently labeled abrin aptamer.

Table S3. Variables Entered/Removed[a].

Model	Variables Entered	Variables Removed	Method
1	x31	.	Stepwise (Criteria: Probability-of-F-to-enter <= 0.050, Probability-of-F-to-remove >= 0.100).
2	x27	.	Stepwise (Criteria: Probability-of-F-to-enter <= 0.050, Probability-of-F-to-remove >= 0.100).
3	x76	.	Stepwise (Criteria: Probability-of-F-to-enter <= 0.050,

			Probability-of-F-to-remove >= 0.100).
4	x58	.	Stepwise (Criteria: Probability-of-F-to-enter <= 0.050, Probability-of-F-to-remove >= 0.100).
5	x6	.	Stepwise (Criteria: Probability-of-F-to-enter <= 0.050, Probability-of-F-to-remove >= 0.100).
6	x54	.	Stepwise (Criteria: Probability-of-F-to-enter <= 0.050, Probability-of-F-to-remove >= 0.100).

a. Dependent Variable: y.

Table S4. Model Summary.

Model	R	R Square	Adjusted R Square	Std. Error of the Estimate
1	0.581a	0.337	0.286	0.11327674552
2	0.779b	0.607	0.541	0.09085538254
3	0.882c	0.778	0.717	0.07134937042
4	0.947d	0.897	0.856	0.05080140971
5	0.973e	0.947	0.918	0.03846296783
6	0.986f	0.972	0.952	0.02952590605

a. Predictors: (Constant), x31

b. Predictors: (Constant), x31, x27

c. Predictors: (Constant), x31, x27, x76

d. Predictors: (Constant), x31, x27, x76, x58

e. Predictors: (Constant), x31, x27, x76, x58, x6

f. Predictors: (Constant), x31, x27, x76, x58, x6, x54

Table S5. ANOVA[g].

Model		Sum of Squares	df	Mean Square	F	Sig.
1	Regression	0.085	1	0.085	6.620	0.023a
	Residual	0.167	13	0.013		
	Total	0.252	14			
2	Regression	0.153	2	0.076	9.250	0.004b
	Residual	0.099	12	0.008		
	Total	0.252	14			
3	Regression	0.196	3	0.065	12.818	0.001c
	Residual	0.056	11	0.005		
	Total	0.252	14			
4	Regression	0.226	4	0.056	21.888	0.000d
	Residual	0.026	10	0.003		
	Total	0.252	14			
5	Regression	0.238	5	0.048	32.236	0.000e
	Residual	0.013	9	0.001		

	Total	0.252	14			
	Regression	0.245	6	0.041	46.798	0.000f
6	Residual	0.007	8	0.001		
	Total	0.252	14			

a. Predictors: (Constant), x31

b. Predictors: (Constant), x31, x27

c. Predictors: (Constant), x31, x27, x76

d. Predictors: (Constant), x31, x27, x76, x58

e. Predictors: (Constant), x31, x27, x76, x58, x6

f. Predictors: (Constant), x31, x27, x76, x58, x6, x54

g. Dependent Variable: y

Table S6. Coefficients[a].

Model		Unstandardized Coefficients		Standardized Coefficients	t	Sig.
		B	Std. Error	Beta		
1	(Constant)	0.052	0.030		1.748	0.104
	x31	0.084	0.033	0.581	2.573	0.023
2	(Constant)	0.073	0.025		2.927	0.013
	x31	0.104	0.027	0.719	3.836	0.002
	x27	-0.077	0.027	-0.537	-2.865	0.014
3	(Constant)	0.071	0.019		3.628	0.004
	x31	0.111	0.021	0.763	5.158	0.000
	x27	-0.080	0.021	-0.557	-3.782	0.003
	x76	-0.058	0.020	-0.416	-2.908	0.014
4	(Constant)	0.060	0.014		4.241	0.002
	x31	0.119	0.015	0.824	7.713	0.000
	x27	-0.075	0.015	-0.520	-4.938	0.001
	x76	-0.094	0.018	-0.671	-5.317	0.000
	x58	0.061	0.018	0.435	3.420	0.007
5	(Constant)	0.066	0.011		6.047	0.000
	x31	0.100	0.014	0.687	7.354	0.000
	x27	-0.079	0.012	-0.550	-6.835	0.000
	x76	-0.078	0.015	-0.558	-5.400	0.000
	x58	0.062	0.013	0.444	4.613	0.001
	x6	0.054	0.019	0.288	2.906	0.017
6	(Constant)	0.078	0.009		8.232	0.000
	x31	0.093	0.011	0.642	8.706	0.000
	x27	-0.091	0.010	-0.630	-9.196	0.000
	x76	-0.086	0.011	-0.609	-7.471	0.000
	x58	0.062	0.010	0.448	6.056	0.000
	x6	0.069	0.015	0.363	4.483	0.002
	x54	-0.027	0.010	-0.202	-2.697	0.027

a. Dependent Variable: y

Table S7. Interaction sites between abrin-M1 and abrin.

Name	Interaction Site	
Abrin-M1	C13、A14、G15、A16、A17、G18、G19、C20、U21(T21)、U22 (T22)、G23、U24 (T24)、G25、G26、U27 (T27)、C28、C31、U32(T32)、U33(T33)、U34(T34)	
	Abrin A-chain	Abrin B-chain
Abrin	LEU28、ARG29、GLY30、GLY31、LEU32、ILE33、HIS34、ASP35、VAL38、LEU39、PRO40、ASP41、THR44、GLN206、VAL209、GLN210、ASP211、LEU232、PHE245、VAL246、CYS247、ASN248、PRO249、PRO250、ASN251	ILE1、VAL2、GLU3、LYS4、SER5、ILE7、SER9、SER10、LYS57、SER58、ASP59、LYS60、GLU96、ILE97、TRP98、ASP99、ASN100、GLY101、THR102、ILE104、ASN105、PRO106、LYS107、SER108、ALA109、LEU110、VAL111、THR127、ASN128、GLU129、TYR130、VAL222、LYS224、TYR230、LEU232、TYR233、ASP234、ASP235

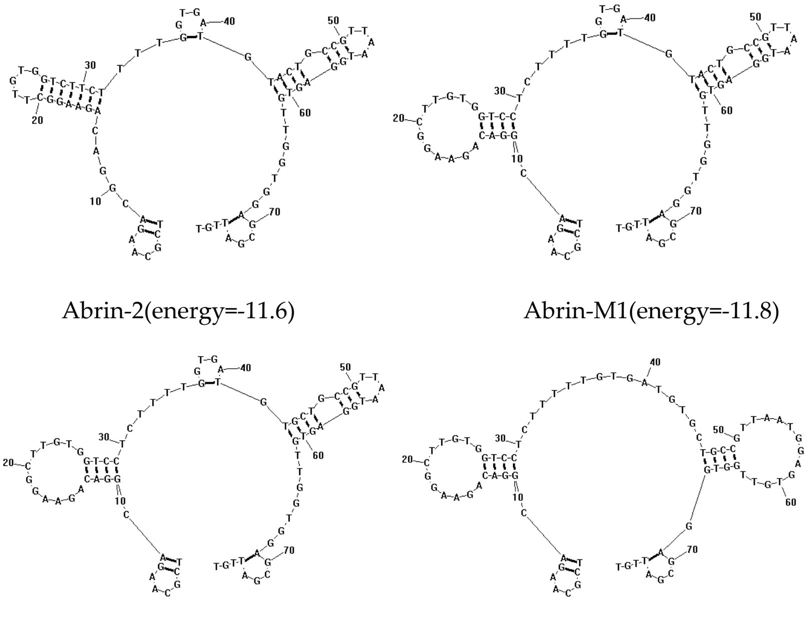

Abrin-2(energy=-11.6) Abrin-M1(energy=-11.8)

Abrin-M2(energy=-10.9) Abrin-M3(energy=-8.8)

Figure S1. Secondary structure of abrin-2 and three new aptamers.

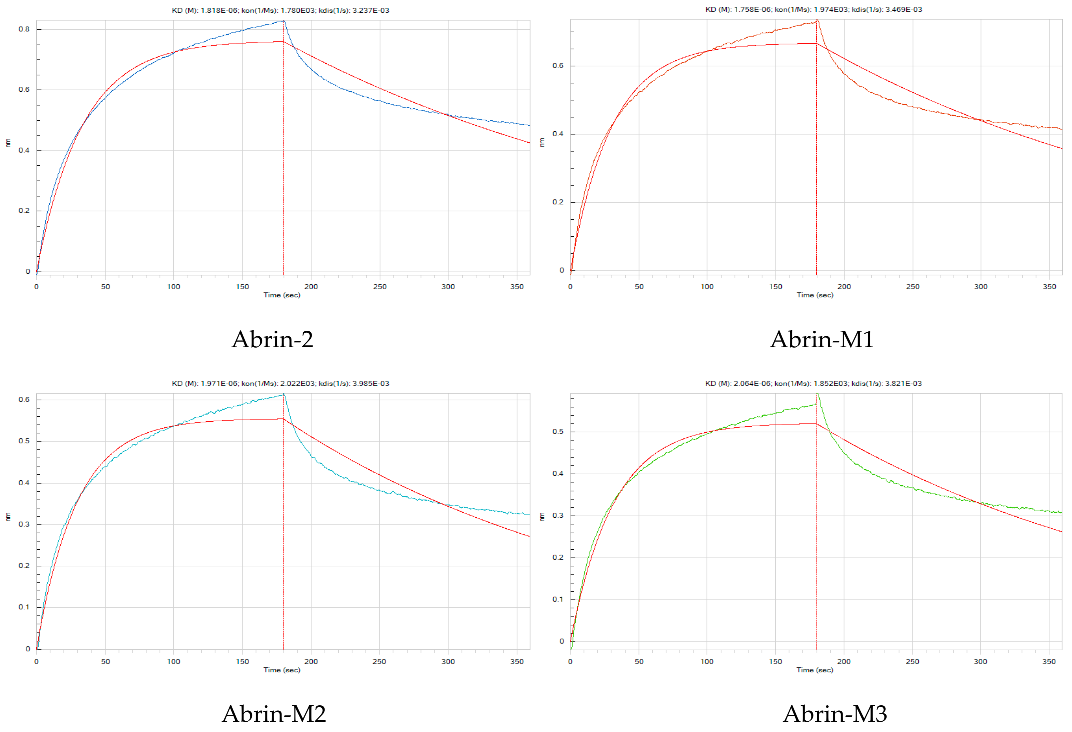

Figure S2. K_D preliminary determination results of abrin-2 and three new aptamers with abrin.

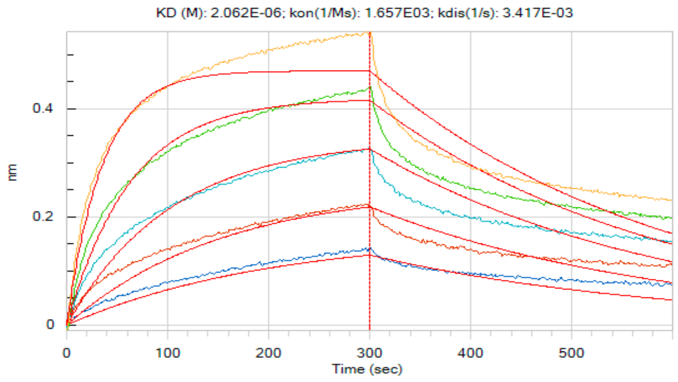

Figure S3. K_D determination results of the ROX labeled abrin-M1 with abrin.
